# Indocyanine green fluorescence in second near-infrared (NIR-II) window

**DOI:** 10.1371/journal.pone.0187563

**Published:** 2017-11-09

**Authors:** Zbigniew Starosolski, Rohan Bhavane, Ketan B. Ghaghada, Sanjeev A. Vasudevan, Alexander Kaay, Ananth Annapragada

**Affiliations:** 1 Edward B. Singleton Department of Pediatric Radiology, Texas Children’s Hospital, Houston, Texas, United States of America; 2 Department of Surgery, Baylor College of Medicine, Houston, Texas, United States of America; 3 Avue LLC., Santa Barbara, California, United States of America; Harvard Medical School, UNITED STATES

## Abstract

Indocyanine green (ICG), a FDA approved near infrared (NIR) fluorescent agent, is used in the clinic for a variety of applications including lymphangiography, intra-operative lymph node identification, tumor imaging, superficial vascular imaging, and marking ischemic tissues. These applications operate in the so-called “NIR-I” window (700–900 nm). Recently, imaging in the “NIR-II” window (1000–1700 nm) has attracted attention since, at longer wavelengths, photon absorption, and scattering effects by tissue components are reduced, making it possible to image deeper into the underlying tissue. Agents for NIR-II imaging are, however, still in pre-clinical development. In this study, we investigated ICG as a NIR-II dye. The absorbance and NIR-II fluorescence emission of ICG were measured in different media (PBS, plasma and ethanol) for a range of ICG concentrations. In vitro and in vivo testing were performed using a custom-built spectral NIR assembly to facilitate simultaneous imaging in NIR-I and NIR-II window. In vitro studies using ICG were performed using capillary tubes (as a simulation of blood vessels) embedded in Intralipid solution and tissue phantoms to evaluate depth of tissue penetration in NIR-I and NIR-II window. In vivo imaging using ICG was performed in nude mice to evaluate vascular visualization in the hind limb in the NIR-I and II windows. Contrast-to-noise ratios (CNR) were calculated for comparison of image quality in NIR-I and NIR-II window. ICG exhibited significant fluorescence emission in the NIR-II window and this emission (similar to the absorption profile) is substantially affected by the environment of the ICG molecules. In vivo imaging further confirmed the utility of ICG as a fluorescent dye in the NIR-II domain, with the CNR values being ~2 times those in the NIR-I window. The availability of an FDA approved imaging agent could accelerate the clinical translation of NIR-II imaging technology.

## Introduction

Indocyanine green (ICG) is a widely used near-infrared (NIR) tricarbocyanine fluorescent dye [1]. Since its approval by the Food and Drug Administration (FDA) in the 1950s [2], ICG has been clinically used in intraoperative angiography for assessment of superficial vessels in the eye [3], coronary artery bypass [4], trauma [5], laparoscopic surgeries [6–8], hepatic clearance [9], aneurysm repair in neurosurgery [10], performing revascularization [11,12] or excluding fistulas [13]. It has been used in the identification and surgical removal of sentinel lymph nodes in breast and skin cancers [14–17]. ICG has also been known to accumulate in tumor cells and lipid-rich plaques in blood vessels, thus facilitating tumor imaging [18] and imaging of atherosclerotic plaques [19]. Clinically approved NIR imaging applications utilizing ICG operate in the first near infrared window (NIR-I, 700–900 nm) with an excitation wavelength of 740–800 nm, and emission wavelength in the 800–860 nm range. Though NIR-I imaging has been very successful in clinical use, it still suffers from poor penetration (~2 millimeters) and a high degree of light scattering [20], thereby resulting in poor spatial resolution.

Recent developments in fluorescence imaging in the second near infrared window (NIR-II), operating in the 1000–1700 nm wavelength range, have shown advantages over NIR-I imaging. Simulation studies using quantum dots in turbid media have suggested that the signal to noise ratio can be greatly improved with fluorophores that emit light at 1320 nm instead of 850 nm [21]. The longer wavelengths utilized in the NIR-II window are advantageous for imaging, as tissue components have reduced photon absorption and scattering effects [20–22], thus enabling deeper tissue penetration, but beyond NIR-II range water absorption becomes dominant, restricting deeper penetration [23,24]. NIR-II imaging has, to date, been impeded by the high cost of sensitive NIR-II detectors. Charge coupled device (CCD) cameras with silicon detectors are generally not sensitive at wavelengths above 1000 nm. Cameras capable of acquiring images in the NIR-II range utilize compound-semiconductor detectors constructed of InGaAs or HgCdTe. These cameras are now becoming affordable and have been engineered to improve quantum efficiency resulting in high contrast sensitivity and resolution [25,26]. The availability of these NIR-II cameras has propelled the development of NIR-II fluorescent dyes and imaging agents for preclinical testing based on a variety of platforms including single-walled carbon nanotubes (SWNTs) [24,27], quantum dot nanoparticles [28,29], rare earth doped nanoparticles [30], polymeric nanoparticles [31–33], and small molecule water soluble dyes [34,35]. NIR-II imaging using these agents have demonstrated improved depth of penetration, thereby enabling sub-surface vascular imaging at high spatial resolution [27,28,35]. The spatial resolution of the proximal femoral artery and vein, in a mouse model, achieved by NIR-II imaging and micro-CT showed that the techniques were comparable in measuring vessel diameters up to several hundred microns [27]. Detection of fluorescence above 1100 nm makes it possible to image through the intact scalp in nude mice at a high resolution [35]. Unfortunately, there are no NIR-II fluorescent agents approved for clinical use. The availability of a FDA-approved NIR-II agent could accelerate the clinical use of this relatively new imaging technique.

In this work, we investigated the fluorescence emission properties of ICG in the NIR-II window. Solutions of ICG were prepared at different concentrations in aqueous and organic media, and evaluated for absorption and NIR-II fluorescence. A custom-built dual camera assembly was developed to facilitate simultaneous imaging of ICG in NIR-I and NIR-II windows. The evaluation of penetration depth was done in vitro by imaging of ICG-filled capillary tubes (as simulated vessels) in Intralipid solution, chicken muscle and calf liver phantoms. In vivo testing was performed in mice injected intra-venously with ICG. The fluorescence properties of ICG were compared with a commercially available small molecule water soluble NIR-II dye, IR-E1050 [36]. Interestingly, we observed that ICG exhibits fluorescence emission in the NIR-II window. In vivo imaging in the NIR-II window enabled visualization of blood vessels with high signal to noise and contrast to noise ratio in comparison to imaging in the NIR-I window.

## Materials and methods

All animal studies were performed under a protocol approved by the Institutional Animal Care and Use Committee of the Baylor College of Medicine. The studies were in compliance with NC3RS-ARRIVE guidelines.

### Absorption and fluorescence emission spectra

The absorption spectra of ICG (Sigma-Aldrich, St. Louis, MO, USA) and IR-E1050 (Nirmidas Biotech, Inc. Palo Alto, CA, USA) were acquired in neutral pH phosphate buffered saline (PBS), ethanol, and plasma. Plasma used in the in vitro studies was reconstituted from lyophilized bovine plasma (Sigma-Aldrich, St. Louis, MO, USA). Measurements were performed at concentrations of 2, 10, and 20 µM. ICG dilutions in PBS and plasma were prepared using a stock solution of ICG (2 mM concentration) prepared in deionized water. ICG dilutions in ethanol were prepared using a 2 mM ICG stock solution made in ethanol. IR-E1050 was obtained as an aqueous solution in PBS at a concentration of ~220 µM. The original solution was diluted in PBS, ethanol, and plasma to obtain the target concentrations. Absorbance spectra of ICG and IR-E1050 dilutions were obtained from 500–1100 nm using a UV-Vis spectrophotometer (UV-1600PC Spectrophotometer, VWR International West Chester, PA, USA). The absorbance spectra were obtained within an hour of preparation of the dilutions. Data acquisition was performed using M-Wave Professional software (version 1.0.20).

The fluorescence emission spectra of ICG and IR-E1050 in the NIR-II window were collected on a NS3 NanoSpectralyzer from Applied NanoFluorescence LLC. (Houston, TX, USA). The emission spectra were collected, for 500 cycles at 10 ms each, using a 782 nm excitation wavelength. ICG dilutions in PBS, plasma, and ethanol were tested. IR-E1050 dilutions in PBS and plasma were tested. Normalized intensity was determined as the ratio of fluorescence emission intensity of ICG (in plasma or ethanol) or IR-E1050 (in plasma or PBS) to that of ICG in PBS.

### In vitro phantom studies for testing fluorescence in NIR-II window

The depth of penetration for NIR-II imaging was tested in: (1) Intralipid^®^ phantom, (2) chicken breast muscle phantom, and (3) calf liver phantom (as a surrogate for highly perfused tissue).

#### Intralipid^®^ phantom imaging

In vitro testing in an intralipid phantom was performed similar to methods described previously [24]. A 1% Intralipid^®^ solution was prepared by diluting 20% Intralipid^®^ (Baxter Healthcare Corp., Deerfield, IL, USA) in deionized water. A cylindrical reservoir filled with 1% Intralipid^®^ solution was positioned on a stage. The height of the stage was adjusted with a micrometer driver (Mitutoyo 151-411ST). A capillary glass tube (OD = 1.55mm/ ID = 1.0 mm) filled with 50 µM ICG in bovine plasma was immersed in the Intralipid^®^ solution. The capillary tube was imaged at depths from 1–5 mm from the top surface.

**Fig 1:**
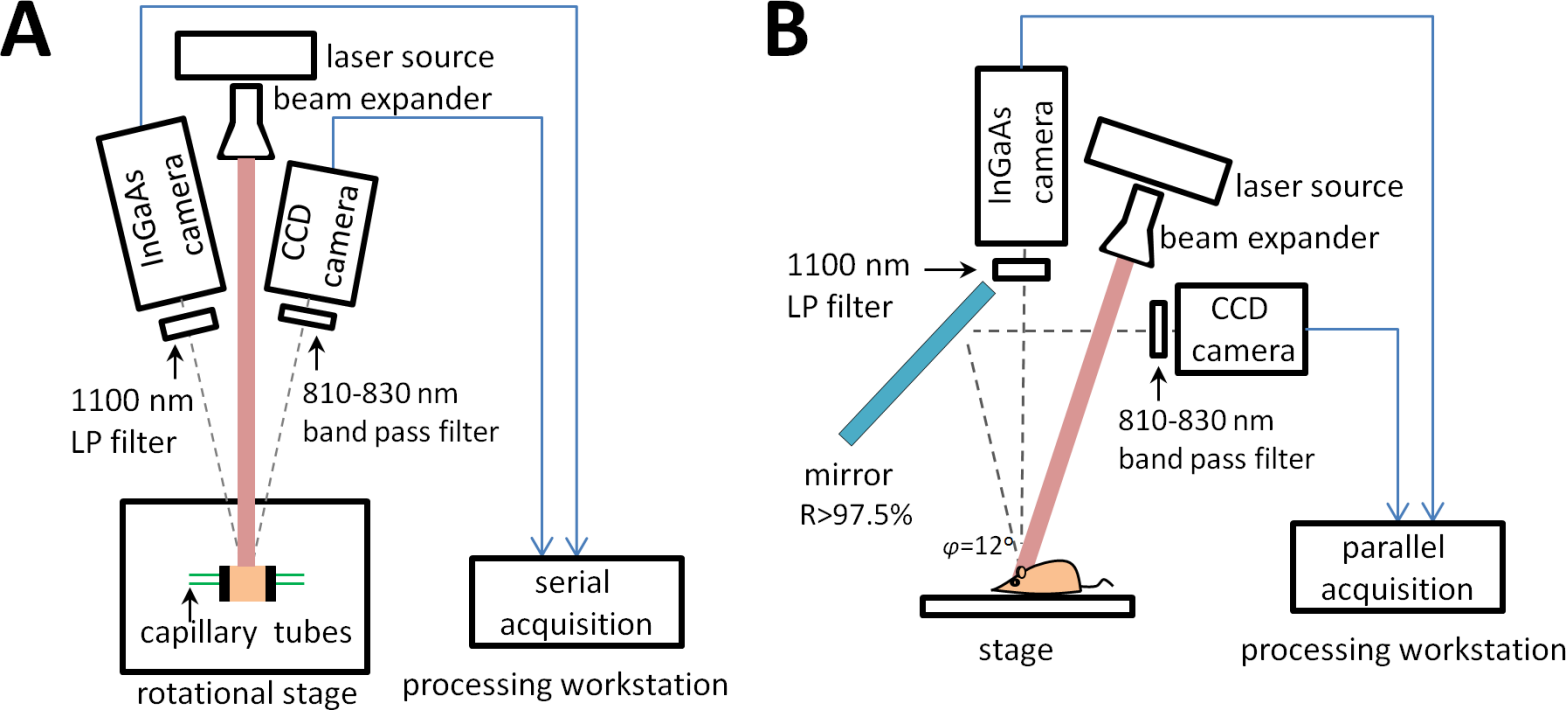
Schematic of imaging setups, (A) imaging setup for tissue phantom experiments (top view), (B) imaging setup for Intralipid^®^ phantom and in-vivo experiments (side view).

A Raptor-Ninox 640 SWIR camera (acquired from Phoenix Engineering Inc., Berkeley Lake, GA, USA) was used to acquire images (50 frames at 40 ms each) in the NIR-II window. A long pass 1100 nm filter (Premium Longpass filter, cut-on wavelength 1100 nm, FELH1100, ThorLabs Inc., Newton, NJ, USA) was placed in front of the camera lens thus restricting wavelengths below, and allowing wavelengths above 1100 nm to pass through the camera lens. Images in the NIR-I window (for ICG) were captured (50 frames at 20 ms each) using a Pioneer camera (Basler, Ahrensburg, Germany), with a Sony ICX625 CCD sensor 2456 x 2058 pixels (5 Mpixels), fitted with 810–830 nm bandpass filter. The imaging setup for the Intralipid^®^ phantom is shown in Fig 1B. All imaging experiments were performed in triplicates.

#### Tissue phantom imaging

Calf liver and chicken breast ware purchased from a local outlet of the Whole Foods national chain of supermarkets (Whole Foods Market, Austin, TX). Two 3D printed plates with 2 mm holes accommodating inserted capillary glass tubes (OD = 1.55mm/ ID = 1.0 mm), were used to hold and position the chicken muscle or calf liver tissue (model of the plate is available on http://www.thingiverse.com/thing:1733616). A schematic of the imaging set-up is shown in Fig 1A. Frozen tissues, cut into cuboids, were placed between two plates and the capillary tubes were inserted through them, through the holes in the plates. The capillary glass tubes were inserted at depths of 3 mm and 6 mm from one edge of the plate. The tissue surface-edges were trimmed to the plate dimensions. The phantom was set up with the surface of the tissue proximal to the tubes facing the camera. The capillary tubes were filled with solutions of either ICG or IR-E1050 in plasma and PBS. To prevent from spills and minimizing the possibility of imaging the residues of previous dye resting in the capillary tubes, new chicken muscle phantoms, and calf liver phantoms were assembled for each dye and each of the three repetitions. The phantoms were excited by the laser source described above using higher settings of 475 mA and 2V, 400 mW optical power. The laser source power density at the imaging stage was 55 mW/cm2, well below the limit of 329 mW/cm2 established in [37] for safe exposure. Images were acquired using the Raptor-Ninox 640 SWIR camera for NIR-II imaging and Pioneer camera for NIR-1 imaging with settings described in Intralipid^®^ phantom imaging section.

### In-vivo testing in NIR-I and NIR-II window

In vivo imaging was performed in nude mice 22 +/- 0.95 g. Anesthesia induction using 2% isoflurane was performed in an induction chamber followed by maintenance on 1–1.5% isoflurane delivered using a nose cone setup. The tail vein was catheterized for injection of the dye. The animals were secured to a platform in the supine position, with the ventral side facing the camera and laser source. The hind limb region of the animal was exposed to the 785 nm laser source and baseline pre-contrast NIR-I and NIR-II images were acquired using the respective cameras. A schematic of the in vivo imaging set-up is shown in Fig 1B. In order to acquire NIR-I images, a gold plated mirror (Mirror FS 1/10 wave gold 100 SQ, Edmund Optics, NJ, USA) was utilized to reflect the image from the animal to the camera lens. ICG injected animals were imaged simultaneously by the NIR-I and NIR-II cameras. Angular difference between collected images ϕ was kept within 12±0.2^o^ range. ICG solution was made fresh and used within 3 hours. The IR-E1050 injected animals were imaged only by the NIR-II camera. Animals (n = 5) were injected with a bolus of either ICG solution in PBS (0.909 +/- 0.04 µmole/kg or 0.681 +/- 0.03 mg/kg) or IR-E1050 in PBS (1 +/- 0.04 µmole/kg or 3.04 +/- 0.13 mg/kg) via the tail vein catheter and dynamic imaging was performed for the hind limb region. The bolus injection of the dyes was followed by a 100 µl saline flush, the procedure not taking more than 5 seconds. Images were captured during injection and at 5, 10, and 15 minutes post-injection. Images at 500 frames at 40 ms were captured by both cameras, the total time for each image acquisition was ~41 secs. The incident laser power used for imaging ICG in the NIR-II window was reduced to 200 mW (half of that used during the NIR-I imaging of ICG and NIR-II imaging of IR-E1050). This was done due to the saturation of signal in the region of interest, at the higher laser power (400mW).

### Data analysis

Image analysis was performed with FIJI software (v 2.0.0-rc-49/1.51f, https://imagej.net/Fiji/Downloads). Image resolution of the NIR-I camera was 2456x2058 pixels compared to 640x512 pixels for NIR–II camera, we compensate the large difference in the resolutions of the images collected with the NIR-I camera by binning them with factor 4, resulting in a 614x514 matrix size. Average intensity image of the 500 images stack was used for analysis. Quantitative analysis of enhancement was performed based on measurements of signal intensities (*SI*) in the manually selected regions of interest (ROIs). Signal-to-noise ratios (*SNR*) were calculated as mean signal intensity in ROIs divided by noise, where noise was represented by the standard deviation (*SD*) of the signal intensity in the air. The placement of ROIs is illustrated in S3 Fig. Contrast to noise ratios (*CNR*) for the tissue phantoms were calculated as a difference between *SNR* of a tube in tissue and *SNR* of the region proximal to tube expressed by the Eq (1).

$${CNR}_{in\;tube}={SNR}_{tube\;in\;tissue}-{SNR}_{\;region\;proximal\;to\;tube}$$ (1)

For the in vivo studies, the CNR was calculated similarly by estimating the SNR in the vessel and SNR in the region proximal to the vessel and subtracting these values as shown in Eq (2).

$${CNR}_{in\;vessel}={SNR}_{vessel}-{SNR}_{\;region\;proximal\;to\;vessel}$$ (2)

A statistical analysis was performed using Kruskal-Wallis to compare medians of calculated CNRs, where the *p*-values are determined based on chi-square statistics.

## Results

### Absorption and fluorescence emission spectra

ICG absorbs light over a broad wavelength range 650–840 nm in PBS, plasma, and ethanol S1A–S1C Fig. In PBS, ICG demonstrated absorption peaks at 710 nm (20 and 10µM) and 780 nm (20, 10, and 2µM). The absorption was higher in plasma and ethanol S1 B and S1C Fig than in PBS S1A Fig, with a peak absorption at ~800 nm in plasma and ~790 nm in ethanol. These findings are consistent with previous studies [38,39], where ICG was shown to exhibit a bimodal absorption spectrum in aqueous media due to the formation of ICG dimers at concentrations as low as 0.1 µM. The higher absorption in plasma is attributed to the adsorption of ICG on to protein molecules in plasma[38].

IR-E1050 absorbs light in the 700–870 nm range with a peak at approximately 780 nm. The peak heights remain unchanged in the three media S1D–S1F Fig. At equivalent concentrations, IR-E1050 demonstrated a lower absorption maximum compared to ICG (~10 times lower).

**Fig 2:**
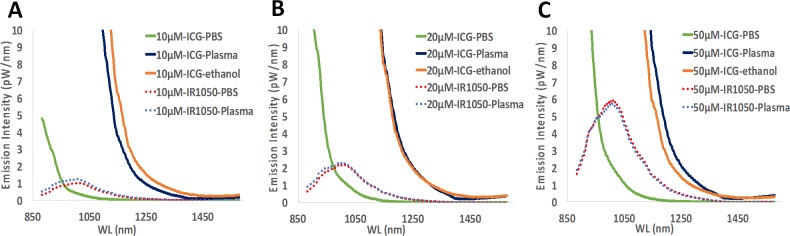
Fluorescence emission profiles of ICG (in PBS, plasma, and ethanol) and IR-E1050 (in PBS and plasma) at (A) 10, (B) 20, and (C) 50 µM.

Fluorescence emission spectra were determined for ICG and IR-E1050 over 880 nm to 1600 nm range (Fig 2). In PBS, IR-E1050 exhibited a higher fluorescence emission compared to ICG in the 1000 nm– 1200 nm wavelength window. However, in plasma and ethanol, ICG exhibited higher emission values in the NIR-II window in comparison to IR-E1050.

The normalized fluorescent intensity (using intensity of ICG in PBS as the normalizing factor) was determined for ICG (in plasma and ethanol) and IR-E1050 (in PBS and plasma) for different emission wavelengths S2 Fig. For emission wavelengths from 900nm– 1150 nm, ICG in plasma and ethanol exhibited higher fluorescence than IR-E1050. The fluorescence values reached up to 3 orders of magnitude higher at 900 nm S2A Fig, and 1–2 orders of magnitude higher at emission wavelengths over 1000 nm S2C–S2F Fig.

### In vitro phantom studies for testing fluorescence in NIR-II window

#### Intralipid^®^ phantom imaging

**Fig 3:**
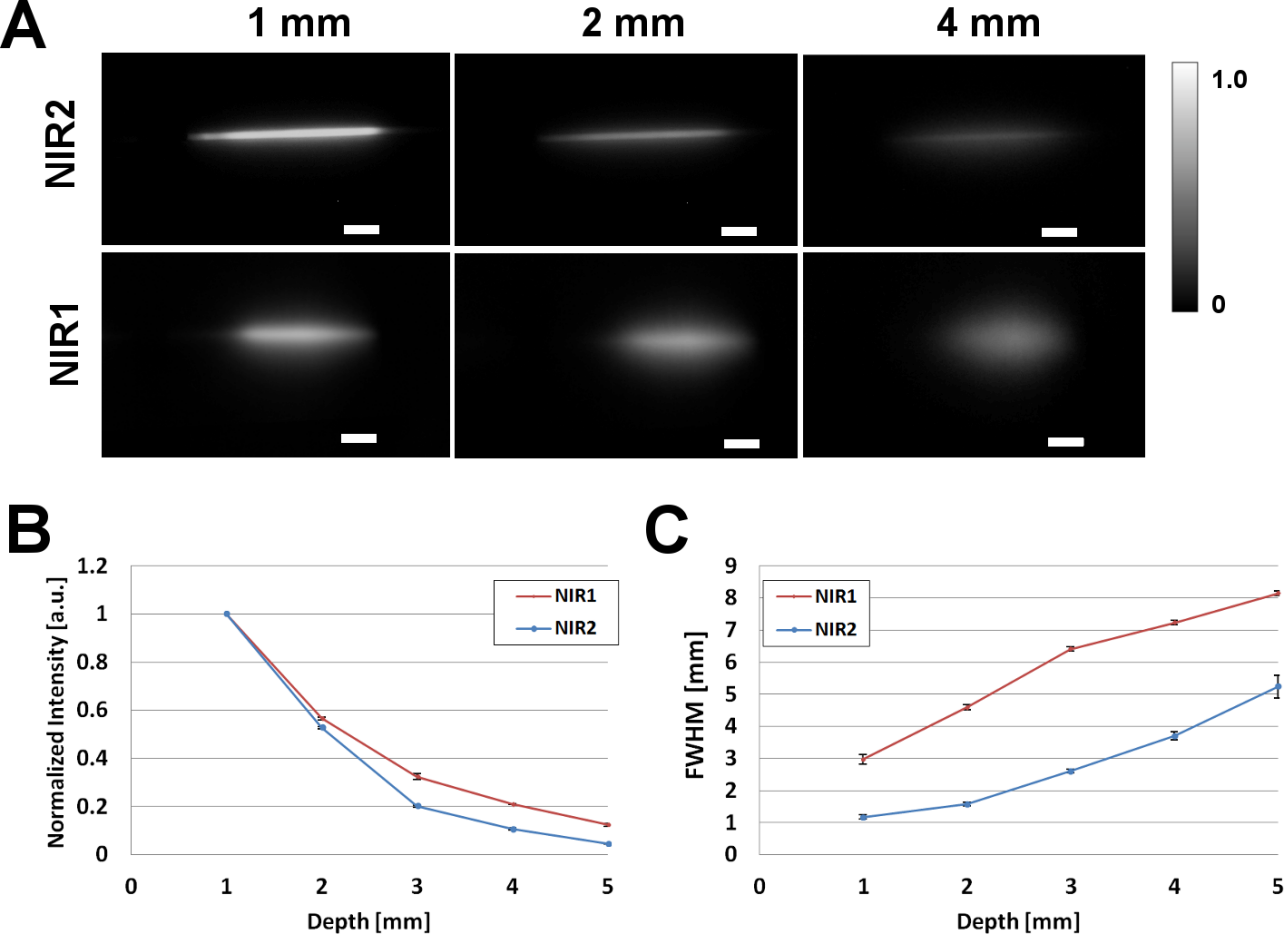
Intralipid^®^ phantom study of ICG in NIR-I and NIR-II window. (A) Fluorescence images in NIR-II (top panel) and NIR-I window of glass capillary filled with ICG in plasma (50µM) at depths of 1, 2, and 4 mm in 1% Intralipid^®^. 785 nm laser used for excitation. Scale bars are 50 mm. (B) Normalized intensity loss of ICG in plasma, in NIR-I and NIR-II as a function of depth. (C) Full-width-half-maximum (FWHM) of capillary glass tube filled with ICG in plasma as a function of depth in Intralipid^®^, showing loss of feature consistency in NIR-I compared to the NIR II. For NIR-I camera standard deviation of the signal intensity in the air was 0, which causes an indefinite value of CNR, for the NIR-I and NIR-II comparisons we plotted both Normalized Intensities and FWHM as in [24]. While for tissue phantoms and in-vivo experiments we report CNR values.

NIR-I and II images of capillary tube filled with ICG in plasma (50µM) immersed in 1% Intralipid^®^, at depths of 1, 2, and 4 mm from the surface are shown in Fig 3A. The edges of capillary tube is clearly visible in the NIR-II window for the three depths with minimal scattering effects. In the NIR-I window, the tube at 1 mm depth is visible, while at 2 and 4 mm the scattering effects are more pronounced. Analysis of the normalized intensities at different depths shows that both NIR-I and II suffer losses in intensities as seen in Fig 3B. The intensity at 5mm depth drops to 0.046 +/- 0.001 for NIR-II and 0.124 +/- 0.004 for NIR-I respectively. Despite the relatively higher loss of normalized intensity in the NIR-II at increasing depths, imaging ICG in the NIR-II outperforms imaging in the NIR-I for the measurement of feature width. This improved performance is attributed to reduced scattering in the NIR-II window when compared to the NIR-I. A full-width-half-maximum (FWHM) analysis done at different depths in the two windows is plotted in Fig 3C. The FWHM analysis of the tube at a depth of 1 mm from the Intralipid^®^ surface measures 1.17 +/- 0.06 and 2.97 +/- 0.08 mm in the NIR-II and NIR-I windows respectively. At a depth of 5 mm from the Intralipid^®^ surface, the tube measures 4.99 +/- 0.35 and 8.15 +/- 0.16 mm in the NIR-II and I windows respectively. The capillary glass tube has an O.D. of 1.55 and I.D. of 1.00 mm. The results obtained in our study are consistent with previous studies of imaging in NIR-II window [24].

#### Tissue phantom imaging

**Fig 4:**
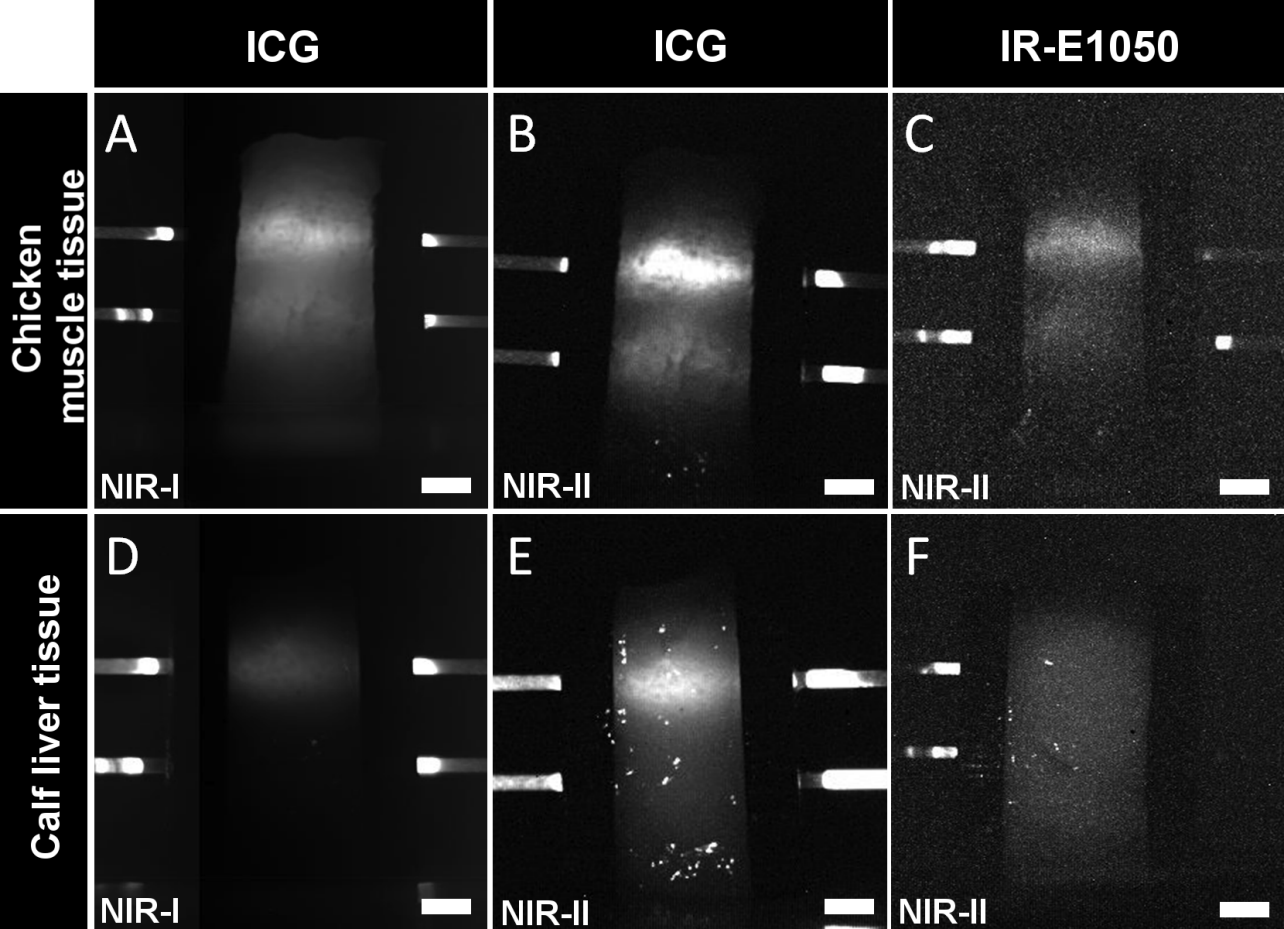
NIR-I and NIR-II images of tissue phantoms with inserted tubes containing ICG or IR-E1050 in plasma. Chicken muscle tissue (*top panel*) ICG in NIR-I (A) and NIR-II window (B). IR-E1050 in NIR-II (C). Calf liver tissue (*bottom panel*), ICG in NIR-I (D) and NIR-II window (E). IR-E1050 in NIR-II (F). Top capillary tube is 3 mm and bottom tube is 6 mm from the surface. Scale bar is 5mm.

**Fig 5:**
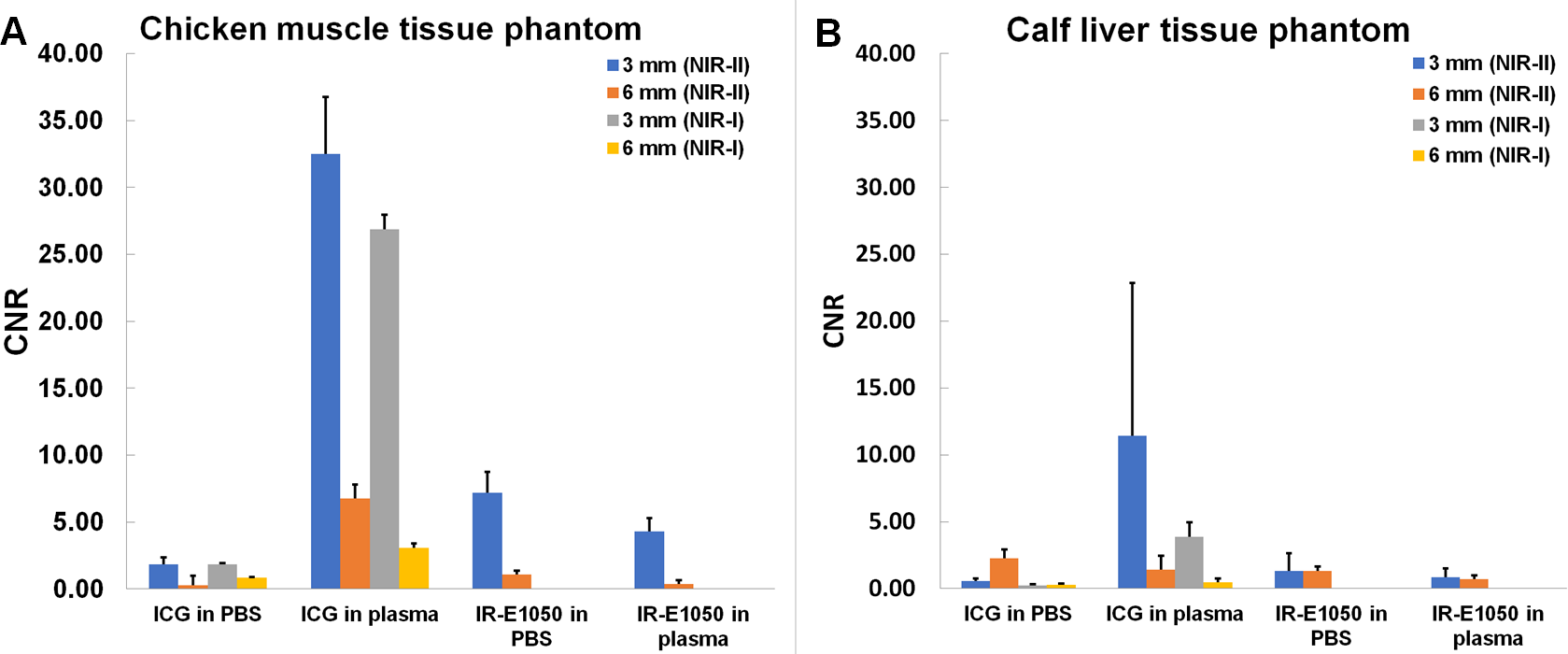
**CNR in capillary tubes embedded at 3 and 6 mm depth from surface in (A) chicken muscle tissue, and (B) calf liver tissue.** Tubes were filled with ICG or IR-E1050 in PBS and plasma and imaged in NIR-I (ICG) and NIR-II (ICG and IR-E1050) window.

The depth of NIR light penetration in tissue was determined in the NIR-I and NIR-II windows using chicken muscle and calf liver tissue. Representative images of the phantoms in the two wavelength ranges are shown in Fig 4. The capillary tubes in the images are filled with the dye (ICG or IR-E1050) in plasma. CNR values for the liver and chicken phantoms are shown in Fig 5. ICG in PBS exhibited low CNR in both the windows in the chicken (CNR of 1.85 +/- 0.57 in NIR-I and 1.82 +/- 0.56 in NIR-II for 3 mm tube) and liver (CNR of 0.24 +/- 0.09 in NIR-I and 0.58 +/- 0.19 in NIR-II for 3 mm tube) tissues. In all cases, ICG in plasma had higher CNRs in chicken (26.86 +/- 5.06 in NIR-I and 32.49 +/- 4.28 in NIR-II) and liver (3.86 +/- 1.1 in NIR-I and 11.43 +/- 11.43 in NIR-II) for 3 mm tube. CNRs in the liver phantoms were generally lower than the corresponding CNRs in chicken phantoms, consistent with the higher blood content and higher scattering in the liver tissue. CNRs for the 6 mm tube in both the windows for the tissues were lower with the exception of the chicken phantom with ICG in plasma (CNR in NIR-II window was 6.77 +/- 3.55 and in the NIR-I being 3.08 +/- 1.66). The CNRs for IR-E1050 were ~5–8 times lower than those for ICG in plasma in the chicken phantom (7.18 +/- 1.57 in PBS and 4.3 +/- 0.98 in plasma for 3 mm tube). The high standard deviations in estimating CNRs for some of the tissues can be attributed to the discrepancies in preparation of a flat surface of the tissue.

### In-vivo testing in NIR-I and II window

**Fig 6:**
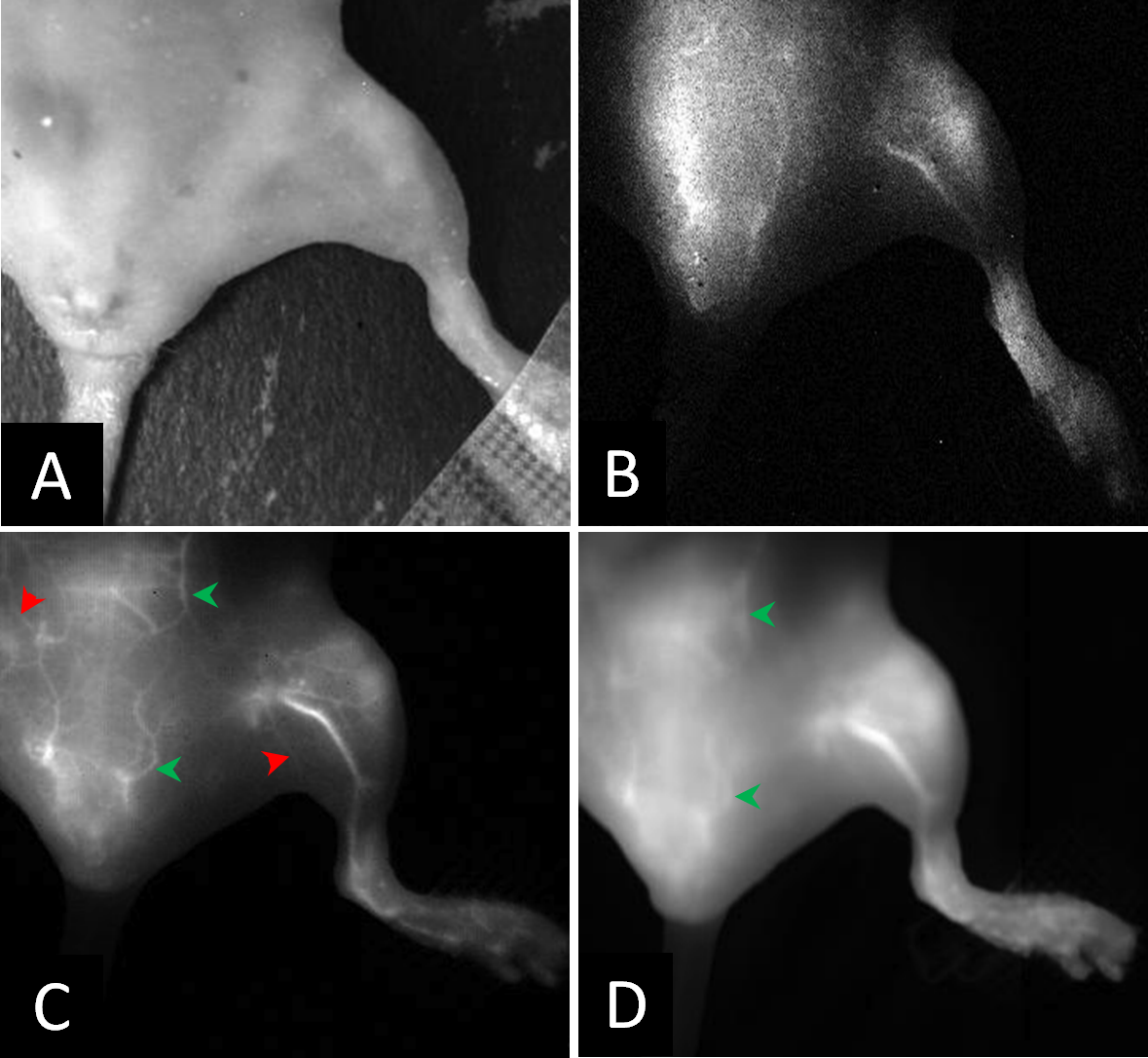
In vivo sub-surface vascular imaging with ICG and IR-E1050. Representative images of the hind limb in a mouse in; (A) visible light, (B) NIR-II window obtained after i.v. administration of IR-E1050, (C) NIR-II window obtained after i.v. administration of ICG, and (D) NIR-I window after i.v. administration of ICG. (C) and (D) is the same animal. NIR images at 5 minutes post injection.

**Fig 7:**
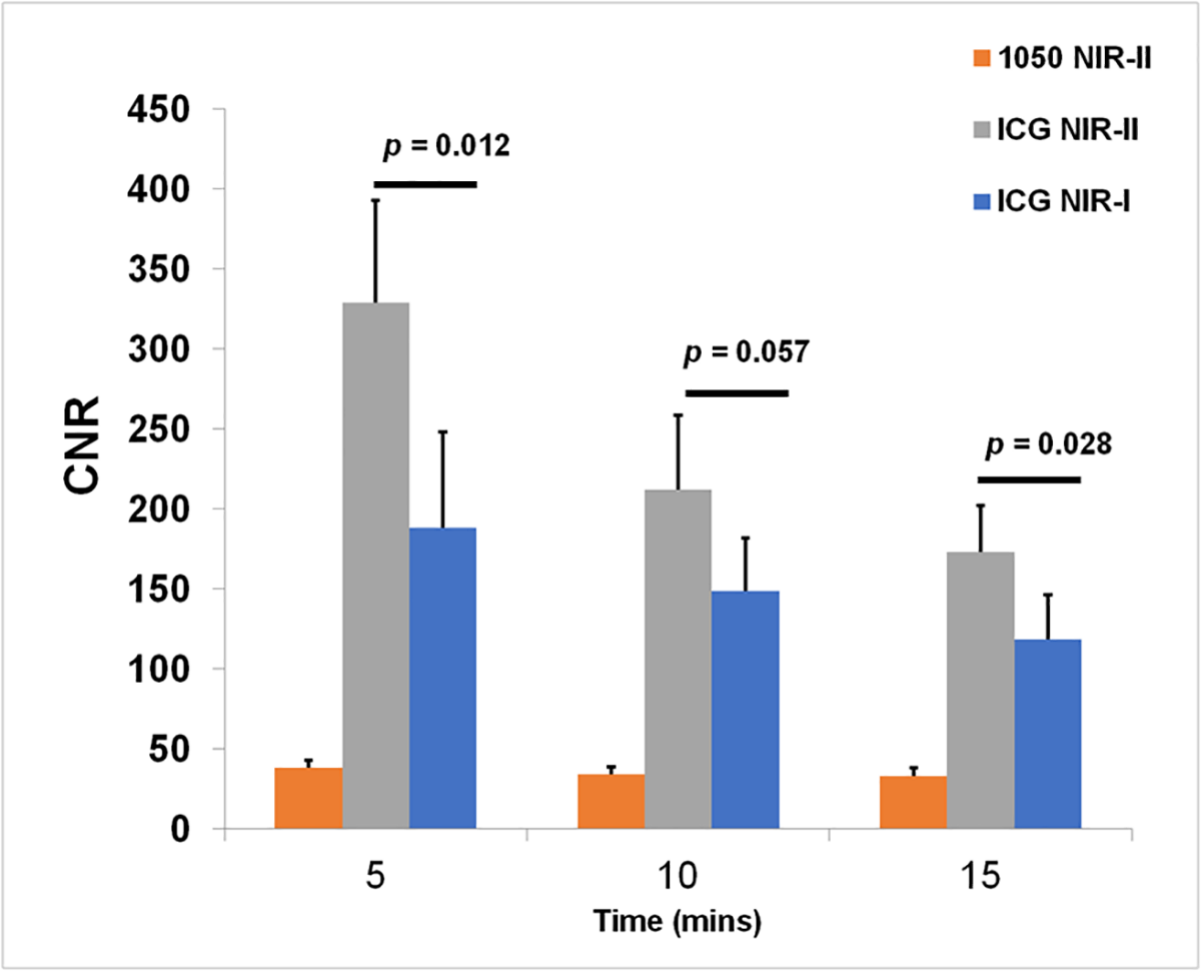
CNR in femoral vessel of animals injected with ICG or IR-E1050 in NIR-I (ICG) and NIR-II (ICG and IR-E1050) window (n = 5).

Imaging of the hind limb in nude mice was done to visualize blood vessels. IR-E1050 injected animals were imaged with the NIR-II camera. For ICG injected animals, simultaneous acquisition allowed for direct comparison of vessel enhancement with NIR-I and NIR-II cameras during injection (S1 Movie and S2 Movie) and at 5, 10, and 15 minutes post-injection. Fig 6 shows representative images of the hind limb in visible light (Fig 6A), IR-E1050 injected in NIR-II (Fig 6B), and ICG injected in NIR-II (Fig 6C) and NIR-I (Fig 6D) window. The NIR images shown were acquired 5 minutes post injection of the dyes. CNRs in the femoral vessel at 5, 10 and 15 minutes post injection are plotted in Fig 7. The CNRs in the NIR-II window following ICG treatment (328.89 +/- 64.24 at 5 mins, 212.06 +/- 46.55 at 10 mins, and 173.14 +/- 29.42 at 15 mins) were ~5–8 times higher than that for IR-E1050 (38.22 +/- 27.11 at 5 mins, 34.18 +/- 21.82 at 10 mins, and 33.37 +/- 22.52 at 15 mins) for an equivalent dose on mole basis. Visualization of vessels with ICG in the NIR-II window was also improved in comparison to NIR-I window, with CNRs ~ 1.4–1.8 times of that in the NIR-I window (188.21 +/- 59.77 at 5 mins, 148.75 +/- 32.92 at 10 mins, and 118.56 +/- 28.18 at 15 mins). Vessels smaller than 200–350 µm, in the lower abdomen region are easily visible in NIR-II (red arrowheads in Fig 6C) but are indistinguishable on NIR-I (Fig 6D). Vessels larger than 350 µm (green arrowheads in Fig 6C) are barely visible in NIR-I (Fig 6D green arrowheads). It should be noted here that the incident laser power used for imaging ICG in the NIR-II window was reduced to half of that used during the NIR-I imaging of ICG and NIR-II imaging of IR-E1050. The laser power was reduced for NIR-II imaging of ICG since the signal was saturated at the higher laser power, making it difficult to visualize the vasculature.

## Discussion

The long wavelength NIR-II window (1000–1700 nm) has recently been proposed as an alternative to the NIR-I window (700–900 nm). The longer wavelengths utilized in the NIR-II window are less affected by photon absorption and scattering effects [20–22] in tissue, thus improving signal-to-noise and depth of penetration [23,24]. To this end, several agents in the form of nanoparticles [24,27–33] or small molecules [34,35] are being developed as NIR-II imaging agents. However, these agents are not yet approved for clinical use.

Indocyanine green (ICG) is a NIR fluorescent agent, already approved for clinical use. Some examples of ICG use in the clinic include angiography, laparoscopic surgery, hepatic function testing, and lymph node identification and removal in cancers [3,6,8–11,15–17]. In this study, we investigated the fluorescence properties of ICG in the NIR-II window.

We observed that ICG has a significant fluorescence emission in the NIR-II window (Fig 2). Notably, in PBS, ICG has very low fluorescence emission above 1000 nm, but in plasma and less polar solvents such as ethanol, ICG exhibits an enhanced emission from 1000–1250 nm. This fluorescence emission is significantly higher than that for the NIR-II dye, IR-E1050, specifically designed for NIR-II imaging. ICG fluorescence emission in the NIR-I window is enhanced in plasma, organic media [38,40,41], and during non-covalent interactions with lipid molecules [42,43]. We speculate that a similar micro-environment interaction mechanism results in the observed NIR-II signal enhancement. Imaging of ICG filled tube immersed at different depths in 1% Intralipid^®^ showed the improved capabilities of NIR-II imaging over NIR-I owing to the reduced scattering effects (Fig 3). In vitro (tissue phantom) and in vivo measurements of CNR (Figs 5 and 7) demonstrate that ICG in the NIR-II window enables superior image quality when compared to ICG imaging in NIR-I window and NIR-II imaging of the purpose-designed NIR-II dye, IR-E1050. Further, this improved CNR was achieved using a 2-fold lower excitation flux than that used for ICG-imaging in the NIR-I window (in vivo studies), suggesting that at equivalent flux, the CNR enhancement would be even greater.

The *in vivo* imaging setup for simultaneous acquisition with NIR-I and NIR-II cameras utilized a gold plated mirror. The gold mirror was selected due to its good reflectance property (> 97.5%), large area of coverage (100 cm^2^) and absence of light loss due to transmission (Fig 1B). However, this setup causes angular difference ϕ between reflected image acquired with NIR-I camera and the image acquired directly by the NIR-II camera. Intra-group differences regarding hind limb volume, skin surface geometric topology, and femoral vessel morphology are likely to have a more profound effect than the difference introduced by the angle ϕ. This is demonstrated from the CNR bar-plots for the in-vivo study (Fig 7) which show the statistically significant difference (Fig 7, p-value = 0.012 at 5 minutes time point) between NIR-I and NIR-II with our current settings using the gold mirror. Bright spots were observed in the liver tissue phantom, visible in the NIR-II imaging (Fig 4E and 4F). These spots were observed on all the individually prepared liver tissue samples (n = 12) used in this study. The ROIs were therefore carefully drawn to exclude these spots to ensure they do not affect quantitative image analysis. A description of the methodology for ROIs positioning is included in the Supplementary Note in S1 File. Our choice of using glass capillaries was based on previously published work [24,43]. While a mismatch between glass capillary and tissue is expected, future studies will consider use of a vessel-mimicking material that matches either chicken muscle or liver tissue refractive index accurately.

The implications of this work are significant. ICG is already approved for clinical use. At a typical clinical ICG dose of 0.6 mg/kg, we demonstrate superior CNR and image quality in the NIR-II window compared to the clinically used NIR-I window, using a clinically approved excitation wavelength (785nm) at safe power levels (27–55 mW/cm2), which is 5–10% of the safe exposure threshold of 329 mW/cm2[37]. Under these conditions, ICG imaging in the NIR-II window enabled clear visualization of several smaller vessels (Fig 6). This suggests that migrating to NIR-II hardware, with no other changes, should result in a dramatic improvement in NIR image quality in the clinic.

## Supporting information

S1 File (PDF)

S1 Fig Absorbance curves for ICG and IR-E1050 in PBS, plasma, and ethanol. (TIFF)

S2 Fig Comparison of normalized fluorescence intensities for ICG (in plasma and ethanol) and IR-E1050 (in PBS and plasma) for the following fixed emission wavelengths. (TIFF)

S3 Fig Representative regions of interest (ROIs) location on tissue phantoms and hind limb images collected with NIR-II camera. (TIFF)

S1 Movie Contrast enhancement of the hind limb in a nude mouse after i.v. tail injection of ICG acquired with NIR-I camera. (AVI)

S2 Movie Contrast enhancement of the hind limb in a nude mouse after i.v. tail injection of ICG acquired with NIR-II camera. (AVI)
