# Supplementary material for: Indocyanine green fluorescence in second near-infrared (NIR-II) window
Source: PLoS One. 2017 Nov 9;12(11):e0187563. doi: 10.1371/journal.pone.0187563 (PMC5679521; doi:10.1371/journal.pone.0187563)
Supplement: S1 File — (PDF) [file pone.0187563.s001.pdf]

Supplementary Material for article “Indocyanine green fluorescence in second near-infrared (NIR-II) window” Zbigniew Starosolski [1], Rohan Bhavane[1], Ketan B. Ghaghada[1], Sanjeev Vasudevan[2], Alexander Kaay[3], Ananth Annapragada[1]

1 Edward B. Singleton Department of Pediatric Radiology, Texas Children’s Hospital, Houston, Texas 77030, USA., 2 Department of Surgery, Baylor College of Medicine, Houston, Texas 77030, USA,3 Avue LLC., Santa Barbara, California, 93105, USA.

Supplementary note: Methodology of positioning the ROIs for phantom and in-vivo experiments

1) ROIs for phantom experiments

For phantom studies red ROIs were placed on the dotted axis which follows capillary tube, placed in the center of the tube in tissue, dimensions of 1x5mm, yellow ROIs of equal dimensions, were placed in proximity to red ROIs positioned below, with the offset equal to OD of the capillary tube (1.5mm) S3 Fig A. If any artifacts are present in these locations, ROIs would be placed to avoid them, by repositioning to other side or by splitting ROI into smaller ROIs and combining them as a sum of smaller ROIs. Blue ROIs were positioned in the corner of the image where there are no artifacts with size 4x red ROI.

2) ROIS FOR IN-VIVO EXPERIMENTS

For in-vivo experiments, ROIs location are presented in S3 Fig B. The inset panel in figure Fig3B shows the positioning of red ROI over measured vessel, length 2.5 mm, with the width equal to vessel diameter, yellow ROI positioned in the proximity of the vessel at a distance equal to vessel width from red ROI, scale bar length 2.5 mm. Blue ROI in main figure Fig3B is placed in the air to estimate noise, scale bar 5mm. Blue ROIs were positioned in the corner where there are no artifacts with size 2.5x5 mm.
