# Supplementary figures and images for: Indocyanine green fluorescence in second near-infrared (NIR-II) window
Source: PLoS One. 2017 Nov 9;12(11):e0187563. doi: 10.1371/journal.pone.0187563 (PMC5679521; doi:10.1371/journal.pone.0187563)

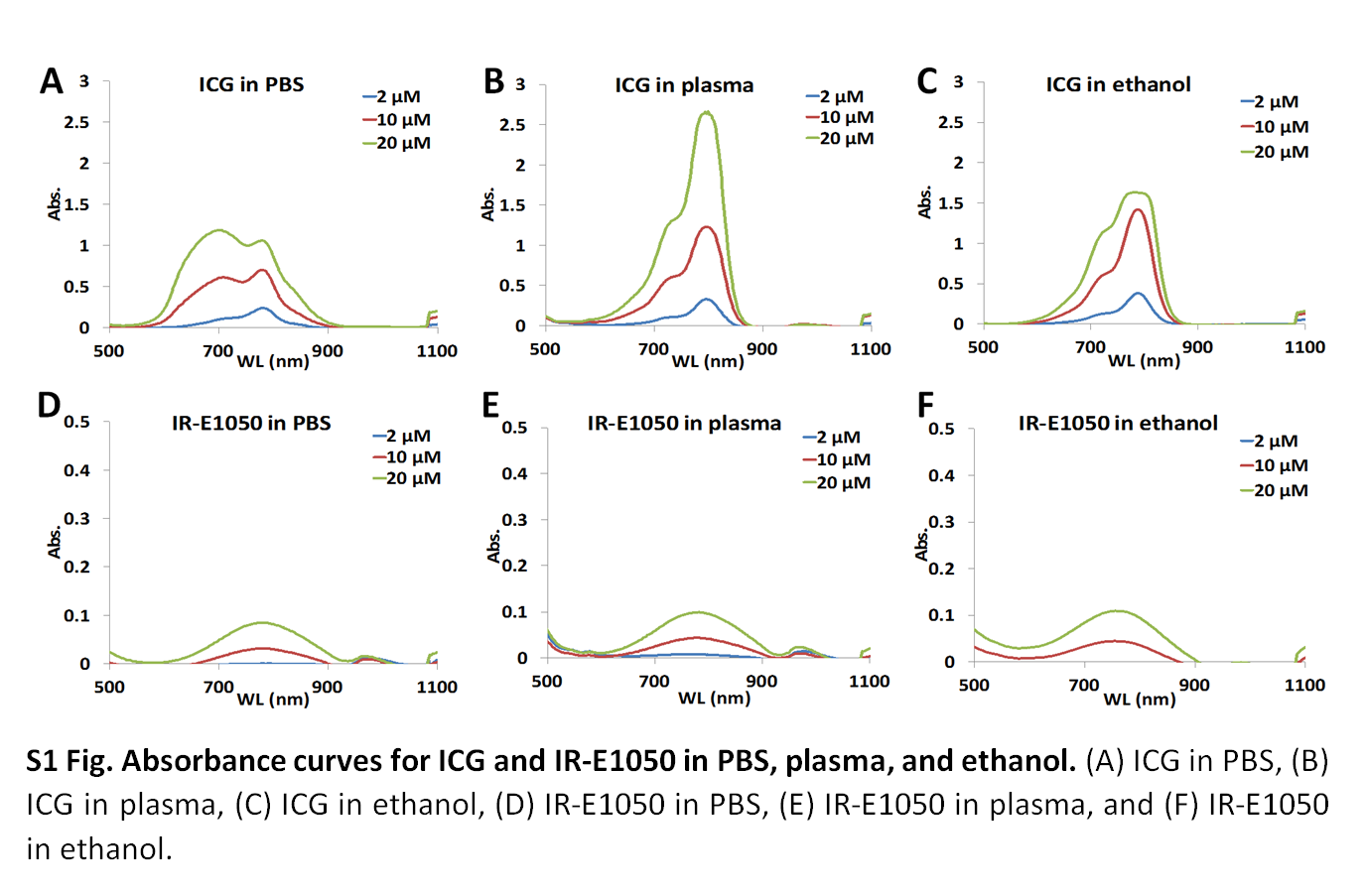

Supplement: S1 Fig — (TIFF) [file pone.0187563.s002.tiff]

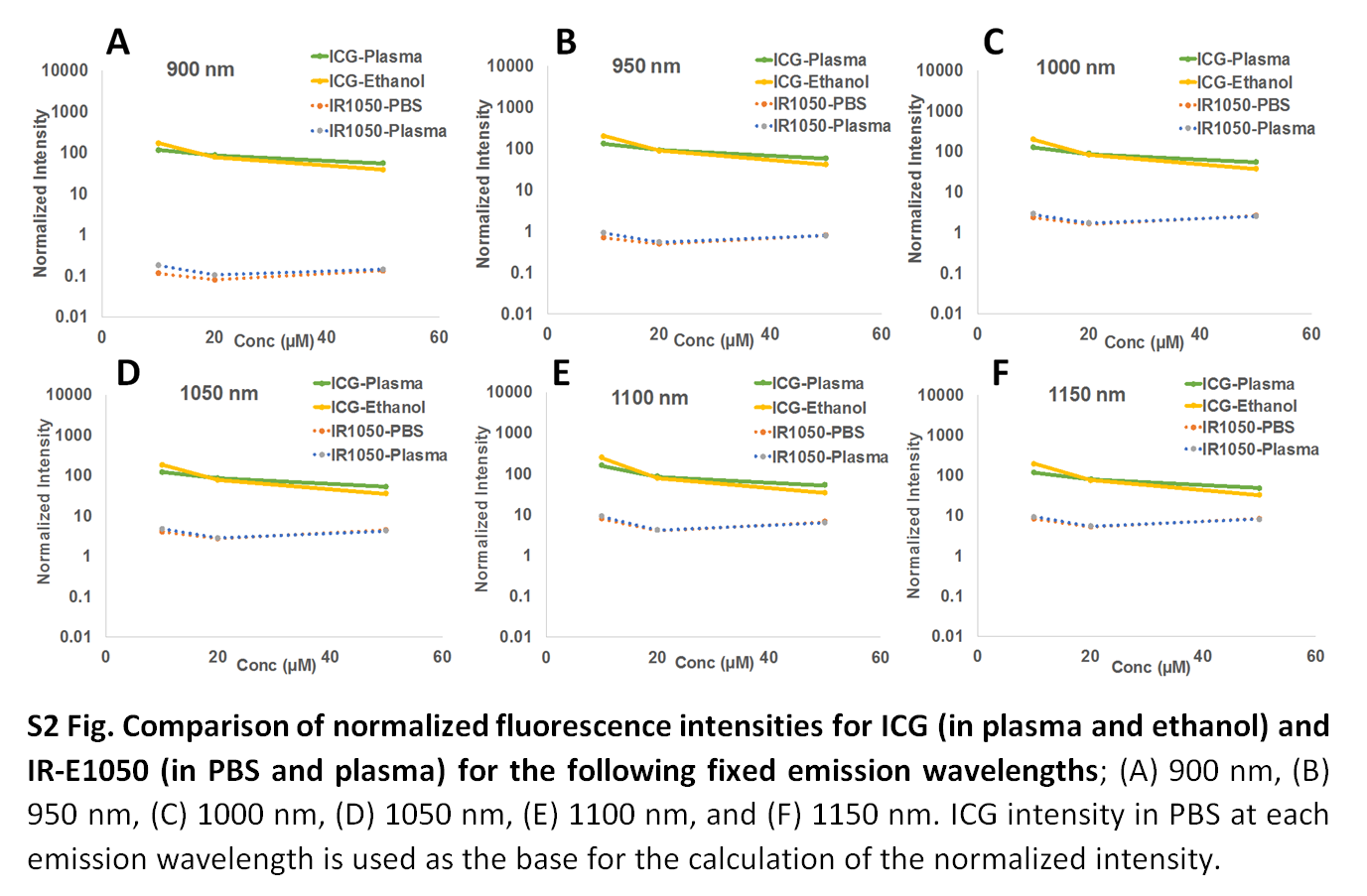

Supplement: S2 Fig — (TIFF) [file pone.0187563.s003.tiff]

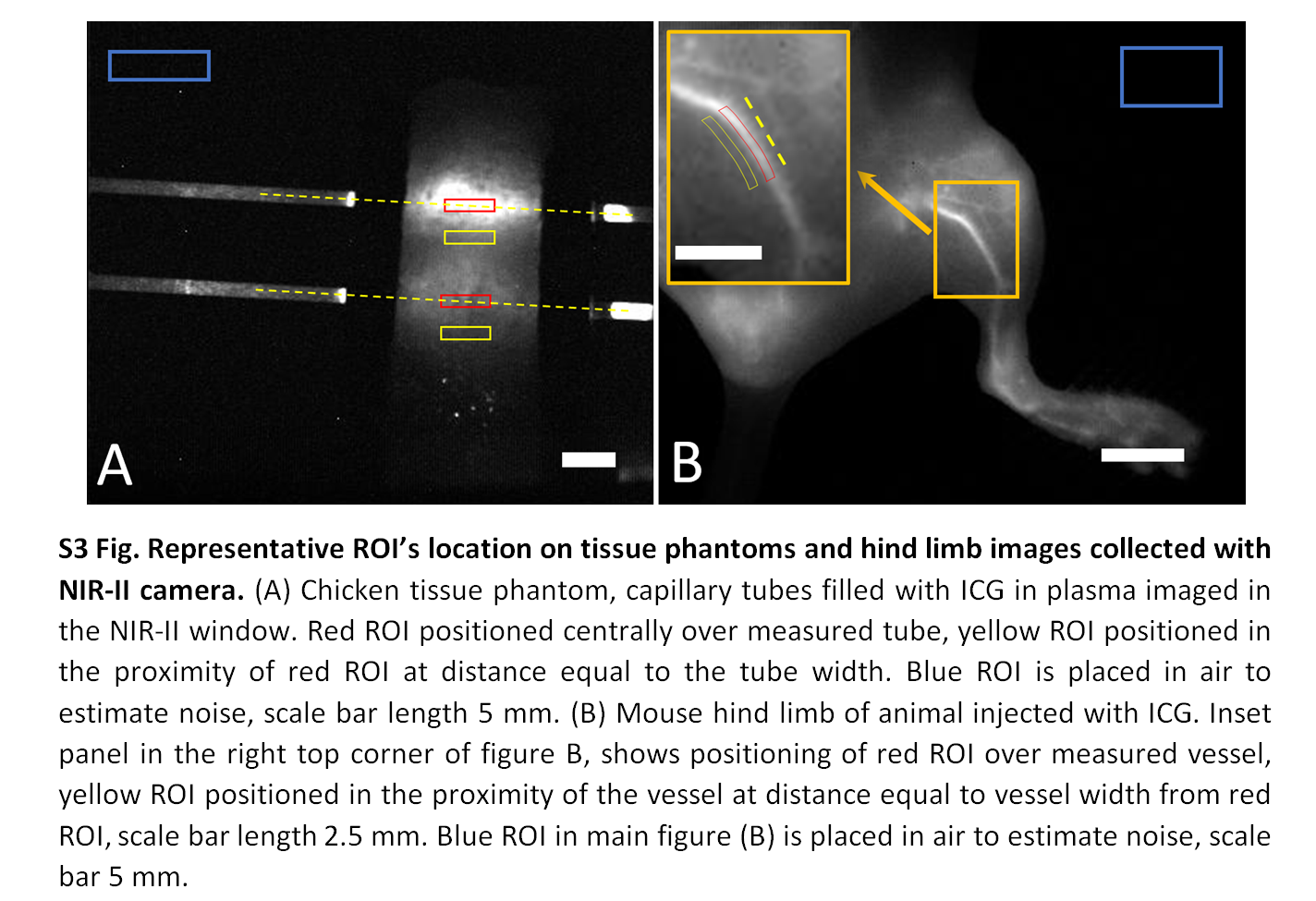

Supplement: S3 Fig — (TIFF) [file pone.0187563.s004.tiff]
